# Enzymatic Synthesis of Trideuterated Sialosides

**DOI:** 10.3390/molecules24071368

**Published:** 2019-04-08

**Authors:** Zhi-P. Cai, Louis P. Conway, Ying Y. Huang, Wen J. Wang, Pedro Laborda, Ting Wang, Ai M. Lu, Hong L. Yao, Kun Huang, Sabine L. Flitsch, Li Liu, Josef Voglmeir

**Affiliations:** 1 Glycomics and Glycan Bioengineering Research Center (GGBRC), College of Food Science and Technology, Nanjing Agricultural University, Nanjing 210095, China; caizhipeng@njau.edu.cn (Z.-P.C.); t2015030@njau.edu.cn (L.P.C.); 2014108037@njau.edu.cn (Y.Y.H.); 2014108004@njau.edu.cn (W.J.W.); wangting@njau.edu.cn (T.W.); 2013208011@njau.edu.cn (H.L.Y.); 2 School of Life Sciences, Nantong University, Nantong 226019, China; pedro.laborda@njau.edu.cn; 3 College of Sciences, Nanjing Agricultural University, Nanjing 210095, China; luaimin@njau.edu.cn; 4 Manchester Institute of Biotechnology, University of Manchester, Manchester M1 7DN, UK; kun.huang-3@manchester.ac.uk (K.H.); sabine.flitsch@manchester.ac.uk (S.L.F.)

**Keywords:** *N*-glycolylneuraminic acid, isotope labeled carbohydrates, sialic acid quantification, sialic acid biosynthesis

## Abstract

Sialic acids are a family of acidic monosaccharides often found on the termini of cell surface proteins or lipid glycoconjugates of higher animals. Herein we describe the enzymatic synthesis of the two isotopically labeled sialic acid derivatives d_3_-X-Gal-α-2,3-Neu5Ac and d_3_-X-Gal-α-2,3-Neu5Gc. Using deuterium oxide as the reaction solvent, deuterium atoms could be successfully introduced during the enzymatic epimerization and aldol addition reactions when the sialosides were generated. NMR and mass spectrometric analyses confirmed that the resulting sialosides were indeed tri-deuterated. These compounds may be of interest as internal standards in liquid chromatography/mass spectrometric assays for biochemical or clinical studies of sialic acids. This was further exemplified by the use of this tri-deuterated sialosides as internal standards for the quantification of sialic acids in meat and egg samples.

## 1. Introduction

Sialic acids, a group of nine-carbon sugars, are widely distributed in the animal kingdom and in pathogenic bacteria [1]. Sialic acids are found as terminal entities at the non-reducing end of glycoconjugates and participate in diverse biological events, including cell recognition, cell-to-cell adhesion, receptor-mediated cell signaling and modulations of the immune system [2,3,4,5]. Whereas a variety of over 40 different types of sialic acids was identified in bacteria, in animals only *N*-acetylneuraminic acid (Neu5Ac), *N*-glycolylneuraminic acid (Neu5Gc), and to a lesser extent 2-keto-3-deoxy-D-glycero-D-galactonononic acid (KDN) were observed [6,7,8]. A peculiar deletion in the coding region of the CMP-Neu5Ac hydroxylase (CMAH) gene prevents the endogenous biosynthetic generation of Neu5Gc in humans [9]. However, Neu5Gc which originates from an animal-derived diet can be found in minute amounts in human epithelial and endothelial cell samples [10,11]. Such dietary-Neu5Gc was described to have immunogenic effects on humans, which is also manifested by the presence of anti-Neu5Gc serum antibodies [12]. To gain a better understanding of the function of sialic acids from foodstuffs, the analysis of sialic acids is required. Based on this consideration, the development of novel methods for quantification of sialic acids seems worth investigating.

Several quantitative analytical methods have been developed and used for the determination of sialic acids; the earliest methods used thiobarbituric acid or resorcinol-HCl for derivatization and for generating colored reaction products, which could be then analyzed spectrophotometrically [13,14]. Although facile and well-established, these methods were hampered if ketoacids such as pyruvic acid are present, which are common metabolites in most biological samples. Furthermore, no distinction between different types of sialic acids was possible by using these methods. In recent decades, various chromatographic and electrophoretic methods have been reported, which were either based on gas chromatography [15], capillary electrophoresis [16], or high-performance liquid chromatography (HPLC) [17] coupled with or without mass spectrometry after derivatization with a chromophore or fluorophore for increased detection sensitivities [18]. The quantification of sialic acids relied either on using external standard calibration curves or in some cases on using unnatural sialic acid analogs as the internal standards [19,20,21,22]. Using internal standards is highly desirable, as varying sample losses during sample handling are rendered irrelevant, which improves the accuracy of the sialic acid quantification significantly. For example, unnatural *N*-propionyl- neuraminic acid (Neu5Prop) was investigated as an internal standard and used for the quantification of the sialic acids in human apolipoprotein E [19] and red meat samples [22]. In this work, we describe the synthesis of isotopically labeled sialic acid standards containing three deuterium atoms as compounds. These compounds and their non-deuterated isotopologues were purified and characterized by mass spectrometric and NMR methods. The 3 Da mass difference between tri-deuterated and non-deuterated sialosides can then be utilized for absolute mass spectrometric quantification by using the deuterated compounds as internal standards. This quantification was exemplified by analyzing sialic acids in meat and egg samples and comparing the obtained results with other sialic acid quantification methods.

## 2. Results

### 2.1. Synthesis

**Scheme 1:**
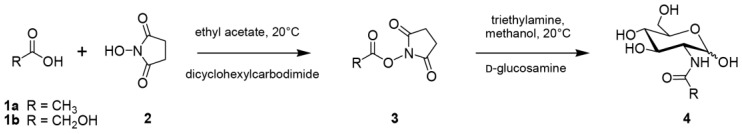
Chemical derivatization glucosamine using activated NHS-acetate and NHS-glycolate to yield N-acetylglucosamine and *N*-glycolyl glucosamine.

In a first step, compounds **4a** and **4b** (Scheme 1), which either bear acetate or glycolate groups, were synthesized using their corresponding *N-*hydroxysuccinimide (NHS) esters and isolated as crude products, yielding 62 and 69 mg, respectively.

**Scheme 2:**
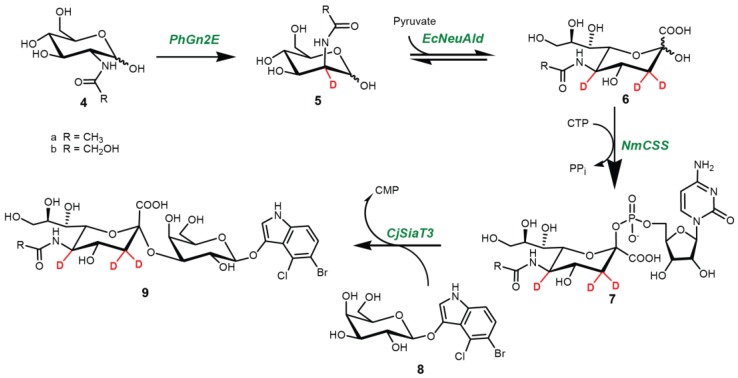
Enzymatic synthesis of d_3_-X-Gal-α-2,3-Neu5Ac (**9a**) and d_3_-X-Gal-α-2,3-Neu5Gc (**9b**). Compound **8** was pre-dissolved in DMF.

Then, a lyophilized enzyme mixture consisting of GlcNAc 2-epimerase (PhGn2E), sialic acid aldolase (EcNeuAld), a CMP sialic acid synthase (NmCSS), α2,3 sialyltransferase (CjSiaT3) was reconstituted in deuterium oxide (D_2_O), and after the addition of pyruvate, CTP, and compounds **4** and **8,** the reaction mixture was typically incubated between 48 h and 96 h at 37 °C (Scheme 2). The same procedure was performed in parallel using H_2_O instead of D_2_O as a reaction solvent. Small aliquots (20 µL) of these 10 mL reactions were used for monitoring the enzymatic conversion from **8** to the sialylated reaction products **9a** or **9b** by HPLC-ESI-MS analysis. In the case of the non-deuterated reaction mixtures, an additional β-galactosidase treatment (EcBGal) was required to remove residual underivatized **8**, yielding free galactose and an insoluble blue indigo dye.

### 2.2. Characterization of Sialosides by HPLC-ESI-MS

**Figure 1:**
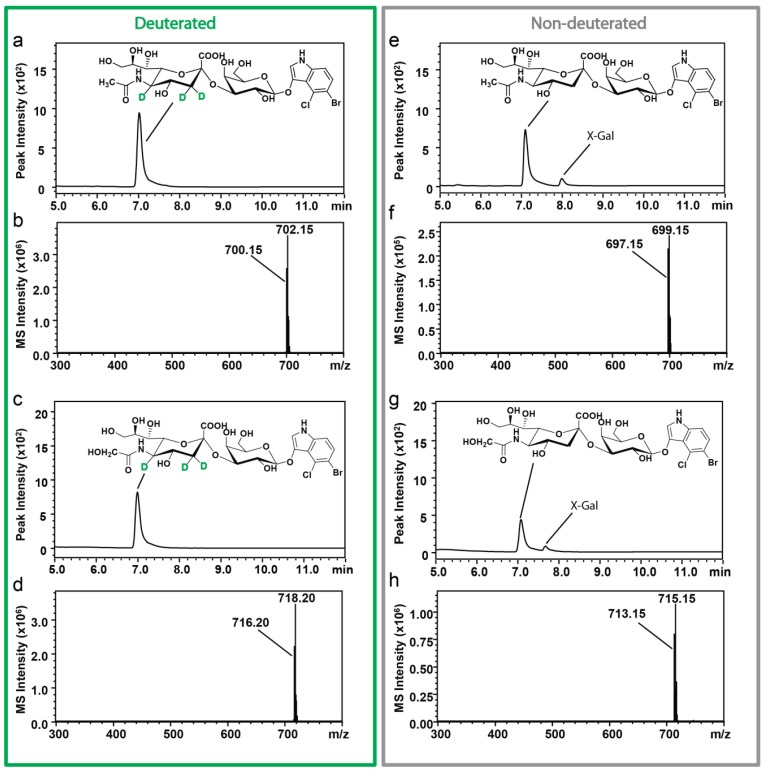
HPLC chromatograms (panels **a**, **c**, **e** and **g**) and ESI-MS spectra (panels **b**, **d**, **f** and **h**) of deuterated and non-deuterated sialosides. The UV absorbance was monitored at 300 nm.

As shown in Figure 1, sialylation of component **8** could be achieved at high conversion rates (for **9a** and **9b**: ~100%; for non-deuterated X-Gal-α-2,3-Neu5Ac and X-Gal-α-2,3-Neu5Gc: 86% and 81%, respectively). The obtained *m/z* signals of negative ion scans were 700.15 and 702.15 for **9a** (Figure 1b), and 716.20 and 718.20 for **9b** (Figure 1d). The observation of two main mass peaks with a mass difference of *m*/*z* = 2 and a smaller mass peak with a mass difference of *m*/*z* = 4 in each mass spectrum can be explained by the almost equal natural abundance of the bromine isotopes ^79^Br and ^81^Br and the distribution of the chlorine isotopes ^35^Cl and ^37^Cl (abundance 76% and 24%, respectively), which are present in the 5-bromo-4-chloro-3-indoxyl aglycone from the X-Gal acceptor substrate. These measured *m*/*z* values correlate well with the expected deprotonated forms of **9a** and **9b** ([M – H]^–^ with isotopes ^79^Br and ^35^Cl calculated: 700.08 and 716.08, respectively). The obtained *m*/*z* signals of the non-deuterated X-Gal-α-2,3-Neu5Ac and X-Gal-α-2,3-Neu5Gc samples were measured at 697.15 Da and 699.15 Da (Figure 1f), and 713.15 Da and 715.15 Da (Figure 1h), which also correlated well with the theoretical *m*/*z* values (([M – H]^–^ with isotopes ^79^Br and ^35^Cl calculated: 697.08 and 713.08, respectively). These values were 3.0 *m/z* units smaller than the corresponding deuterated components **9a** and **9b**, indicating that three deuterium atoms were incorporated in the sialic acid moiety during the enzymatic synthesis from **4** when the reaction took place in D_2_O. These components were then further characterized using NMR spectroscopy.

### 2.3. Characterization of Sialosides by NMR

To fully elucidate which protons in the sialosides were replaced by deuterium atoms, one-dimensional ^1^H- and ^13^C-NMR spectra, and two-dimensional COSY and HSQC spectra were performed (Tables S1 and S2, Figures S1–S16). As shown in Figure 2, a clear distinction between the sialosides synthesized in D_2_O or H_2_O was observed. Proton signals correlating to the position 3 (both axial and equatorial) and 5 of X-Gal-α-2,3-Neu5Ac were significantly reduced (panel a), or in the case of Neu5Gc essentially missing (panel b), when D_2_O was used instead of H_2_O during the biotransformation reaction.

### 2.4. Application of d_3_-Sialosides as Internal Standards

**Scheme 3:**
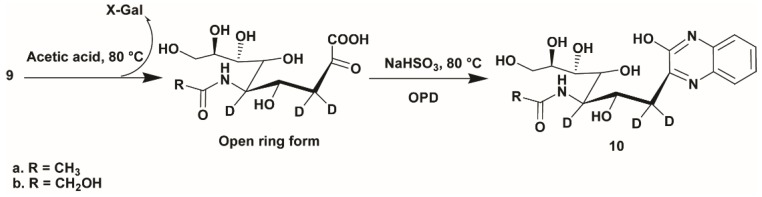
Derivatization procedure of d_3_-sialosides with *O*-phenylenediamine.

In order to test the practical use of the trideuterated sialic acid probes, the synthesized sialosides were subjected to mild acid hydrolysis, and the released free sialic acid consequently labeled with *O*-phenylenediamine (OPD, Scheme 3). The OPD-derivatized components **10a** and **10b** were then analyzed and HPLC-ESI-MS (Supplementary Figure S17). The *m/z* values of samples **10a** and **10b** of 407.0 and 423.0 obtained in positive scan mode correlated well with the expected *m/z* values of the [M + Na]^+^ adducts of **10a** and **10b** (407.16 and 423.16 Da, respectively, Supplementary Figure S17a,b). As expected, hydrolysis and OPD-derivatization of the non-deuterated sialosides resulted in the detection of a 3.0 Da smaller *m*/*z* ratio (404.0 Da and 420.0 Da, Figure S17c,d). The *d_3_-*sialosides were also subject to mild acid hydrolysis and OPD-derivatization in various concentrations of *d_3_-*sialosides **9a** and **9b** (0.015, 0.03, 0.06, 0.15, 0.3, 0.6, and 1.5 mM in the case of **9a** and 0.025, 0.05, 0.1, 0.25, 0.5, 1, and 2.5 mM in the case of **9b**) for obtaining more information on the quantitative response of the signal intensities from the mass spectrometric analysis. For both components, **10a** and **10b**, a quasi-linear relationship between signal intensities from the HPLC-ESI-MS analysis and the sample concentrations were observed (Figure S18). These data were then also used as a calibration curve when quantifying the sialic acid contents in meat and egg samples.

**Figure 3:**
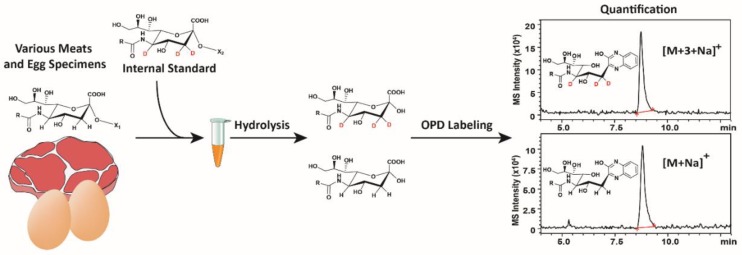
Overview of analyzing the sialic acid concentrations in meat and egg samples using d_3_-sialosides **9a** and **9b** as internal standards.

As shown in Figure 3, the components **9a** and **9b** were then used as internal standards to measure the sialic acid contents of a selection of meats and eggs from various animals using HPLC-ESI-MS analysis. The results showed that the Neu5Gc concentration in meat samples vary significantly between the tested breeds and species, with concentration ranging from 5.7 ± 1.1 µg Neu5Gc/g meat for pork to 22.3 ± 4.0 µg Neu5Gc/g meat for Mongolian mutton (Table 1).

In eggs, the measured Neu5Ac concentration was higher in the egg yolk than in the egg white in all measured samples, with the highest Neu5Ac concentrations measured in pigeon eggs (1.50 ± 0.16 mg Neu5Ac/g egg white and 2.25 ± 0.13 mg Neu5Ac/g egg yolk), and the lowest measured in Grey goose eggs (0.05 ± 0.00 mg Neu5Ac/g egg white and 0.55 ± 0.06 mg Neu5Ac/g egg yolk, Table 2).

## 3. Discussion

### 3.1. Synthesis and Structure Determination of d_3_-Sialosides

In this work, a one-pot four-enzyme reaction which took place in D_2_O as a reaction solvent was carried out to synthesize **9a** and **9b**. A comparable biosynthetic approach (but using H_2_O instead of D_2_O) was recently applied by Both et al. for the synthesis of X-Gal-α-2,3-Neu5Ac and X-Gal-α-2,6-Neu5Ac, which were then used in the biochemical characterization of a linkage specific α-2,6-sialidase [23]. By replacing H_2_O with D_2_O, the mechanistic action of the first two enzymes in this reaction cascade, GlcNAc 2-epimerase, and sialic acid aldolase, allowed the exchange of up to three protons with deuterium atoms: in a recent work, we showed that the epimerization reaction of GlcNAc 2-epimerases is based on a deprotonation/reprotonation mechanism, which allowed the exchange of a single proton at the C2 position of GlcNAc or ManNAc during the epimerization reaction [24]. As we demonstrated in this study, two more deuterium atoms can be exchanged during the sialic acid aldolase-catalyzed aldol-addition: The catalytic reaction mechanism of this enzyme requires one water molecule for the proton transfer from the ManNAc aldehyde, and a second one for the reprotonation for sialic acid release after C-C bond formation from the catalytic lysine residue of the enzyme [25]. No further deuterium atoms were introduced during the activation step by CMP sialic acid synthase (generating the sugar nucleotide CMP-sialic acid), and the sialylation step by α2,3 sialyltransferase (which transfers the sialic acid to the acceptor substrate X-gal). Importantly, it seemed that the overall activity of the four enzymes was not hampered by replacing H_2_O with D_2_O, which indicates the robustness of this enzymatic cascade when D_2_O is used as a reaction solvent.

HPLC-ESI-MS analysis and NMR spectroscopy allowed the elucidation of the structure of the obtained sialosides and confirmed the incorporation of deuterium at the positions C3 (two atoms) and C5 (one atom) of the sialic acid moiety. Furthermore, the substitution with deuterium also caused changes in the chemical shift of several signals; the signal corresponding to S4 in the ^1^H NMR spectra of **9a** and **9b** appeared shifted after the substitution. It must be noted that S4 is located between the two substituted positions S5 and S3. Interestingly, the S4 signal was found to be slightly deshielded (from 3.74 to 3.83 ppm) in compond **9a**, whereas the same signal appeared slightly shielded (from 3.90 to 3.82 ppm) in compound **9b**. In the ^13^C-NMR spectra of **9a** and **9b**, only carbons S3 (which are attached to the deuterium) were significantly shifted. Thus, the signal of carbon S3 in **9a** shifted from 42.23 to 28.20 ppm after the substitution, whereas the signal of carbon S3 in **9b** shifted from 41.92 to 24.23 ppm.

Using X-Gal as acceptor substrate for the sialylation reaction allowed to monitor the progress of the reaction significantly, given the strong UV absorbance of the indoxyl group. This group also allowed us to isolate the reaction product by a single solid phase extraction step using octadecylsilane-bonded silicate (C18) cartridges. In reaction mixtures which could not reach complete sialylation, the residual X-Gal could be simply removed by treating the reaction mixture with β-galactosidase. The released 5-bromo-4-chloro-3-indole then spontaneously dimerized (in the presence of oxygen) into an insoluble blue indigo dye, which can be simply removed by centrifugation.

Although X-Gal was beneficial during monitoring the conversion rates and for the isolation of the reaction products, it’s use as a substrate had also a downside: For the mass spectrometric analysis of the compound, the natural distribution of the containing chlorine and bromine isotopes of the 5-bromo-4-chloro-3-indoxyl portion somewhat complicate the overall mass spectra, as always two main peaks with the mass difference of 2 Da and a smaller mass peak with a mass difference of additional 2 Da were observed [26]. However, in case of the herein described sialylation method the expected sialosides will incorporate three deuteriums, and therefore the overall mass increase is 3 Da, which allows the discrimination from the 2 Da and 4 Da added by bromine and chlorine isotopes. In hindsight, choosing alternative acceptor substrate such as 4-nitrophenyl-β-galactoside or 4-methylumbelliferyl-β-galactoside, which are also commercially available, would have been a valid option and will be considered for future preparations of deuterated sialosides.

### 3.2. Sialic acid Quantification

Using isotopically labeled carbohydrates allows the absolute quantification of natural glycan species. A recent example using isotopically labeled *N*-glycan standards was reported by Echeverria et al., in which a chemically synthesized *N*-glycan heptasaccharide, in which the *N*-acetyl moieties of four GlcNAc units contained ^13^C and resulted in an overall mass increase of 8 Da over the natural isotopologues [27]. This compound was then applied for the absolute quantification of the N-glycan concentration of a therapeutic antibody. Further applications of the herein described enzymatic deuteration method could be also applied to generate libraries of *N*-glycans containing tri-deuterated sialic acids, or mono-deuterated GlcNAc moieties which could be enzymatically generated by using ManNAc as a substrate for epimerization reactions.

Although previously described internal standards (such as *N*-propionylneuraminic acid [19,22]) have no obvious disadvantage over the deuterated samples described in this work (Neu5Ac and Neu5Gc), more challenging sample compositions will benefit from isotopically labeled internal standards; for example, the use of *N*-propionylneuraminic acid may be unsuitable when samples consist of more than two types of sialic acids inseparable from the analytes (i.e., bacterial isolates [28]). Another advantage is that one can expect closely related physico-chemical properties between isotopologues, which is benificial for quantifying sialosides which are more sensitive to acid treatments (i.e., KDN hydrolyzes significantly faster in mild acids compared to Neu5Ac or Neu5Gc [6]).

The herein measured Neu5Gc concentrations of the analyzed meat samples were in good agreement with data from literature reports [10,22]. Mutton and beef generally show higher Neu5Gc contents when compared to pork or game. However, the Neu5Gc concentration in meats varies significantly between individual samples, which may be based on the age and other unknown factors (unpublished data).

The Neu5Ac concentration of the tested egg samples showed a wide variation across species, and are broadly in agreement with published data. For example, Juneja et al. reported 0.95 mg/g of Neu5Ac in the yolk, and 0.1 mg/mL in the egg white of chicken eggs (we measured 1.37 ± 0.15 mg/g and 0.40 ± 0.10 mg/g respectively) [29]. Koketsu et al. presented comparable Neu5Ac concentrations to ours for Silkie egg white in their study [30] However, their work showed a thirteen times higher Neu5Ac concentration in Silkie egg yolks when compared to our measurements, which perhaps merits further studies on the intra-species variation of the sialic acid contents of these eggs in future.

## 4. Materials and Methods

### 4.1. Reagents

d-Glucosamine·hydrochloride and 5-bromo-4-chloro-3-indolyl-β-d-galactopyranoside (X-Gal, compound **8**) were purchased from Aladdin (Shanghai, China); *Pedobacter heparinus* GlcNAc-2-Epimerase (PhGn2E), *E. coli* Sialic Acid Aldolase (EcNeuAld), *Neisseria meningitidis* CMP-Sialic Acid Synthase (NmCTT), *Campylobacter jejuni* Sialyltransferase (CjSiaT3), and *E. coli* β-Galactosidase were obtained from Qlyco Ltd. (Nanjing, China). Acetonitrile and methanol used for HPLC-ESI-MS analysis, and deuterated water and methanol were purchased from Merck (Nanjing, China). All other chemicals used were of the highest grade available.

### 4.2. Chemical Synthesis of N-Acetyl-D-Glucosamine (GlcNAc) and N-Glycolyl-D-Glucosamine (GlcNGc)

The syntheses of GlcNAc and GlcNGc from glucosamine were based on the method described by Lapidot [31]. The procedure for the synthesis of GlcNAc was performed as follows: acetic acid (0.286 mL, 5 mmol) was added to a solution of *N*-hydroxysuccinimide (0.575 g, 5 mmol) in dry ethyl acetate (23 mL). A solution of dicyclohexylcarbodiimide (1.03 g, 5 mmol) in ethyl acetate (2 mL) was then added. The reaction mixture was stirred at room temperature overnight under a nitrogen atmosphere. Then, the precipitate of the reaction mixture was removed by centrifugation at 13,400× g for 10 min. The supernatant contained an NHS-ester of acetic acid was dried by rotary evaporation under reduced pressure and re-dissolved in dry 17 mL of methanol. d-Glucosamine hydrochloride (0.862 g, 4 mmol) and triethylamine (0.56 mL) were then added and dissolved by placing the sample in a heated sonication bath (50 °C for 1 h). The reaction mixture was then stirred at room temperature for 16 h. The sample was again dried using rotary evaporation and washed three times with ethyl acetate (1 mL) to remove the unreacted starting material. After vacuum drying of the sample using centrifugal evaporation, the product was dissolved in 8 mL of 10% methanol in water (*v*/*v*). The supernatant was collected and concentrated using a rotary evaporator under reduced pressure. For the synthesis of GlcNGc, glycolic acid (0.3 mL, 5 mmol) was used instead of acetic acid. As judged by TLC analysis, the conversion of d-glucosamine to the reaction products **4a** or **4b** reached approximately 70%. These amidated reaction products were then used directly as starting material for the enzymatic sialylation reactions.

### 4.3. One-Pot Four-Enzyme Synthesis of d_3_-Sialosides

The synthesis of **9a** was based on a procedure described previously [23,32], with slight modifications and performing the enzymatic reaction in D_2_O instead of H_2_O; in brief, the one-pot four-enzyme reaction was carried out using a lyophilized mixture of MES buffer (50 mM, pH 6.5) contained GlcNAc (10 mmol), CTP (0.15 mmol), pyruvate (0.25 mmol), MgCl_2_ (0.05 mmol), PhGn2E (5 mU), EcNeuAld (5 mU), NmCTT (5 mU) and CjSiaT3 (5 mU). The mixture was dissolved in D_2_O (50 mL), and then X-Gal (0.1 mmol, dissolved in 0.5 mL of DMF) was added. The reaction mixture was then incubated at 37 °C for 48–96 h until more than 80% product formation was observed (based on the amount of X-Gal; HPLC analysis was performed with 20 µL sample aliquots). In cases were no complete conversion was observed, residual X-Gal was removed by adding *E. coli* β-galactosidase (10 U). The supernatant was concentrated and purified using solid phase extraction (RP-C18, Supelco, Bellefonte, PA, USA, 500 mg resin fill, yielding 56 mg of sialoside). For the synthesis of **9b**, GlcNGc (10 mmol) was used instead of GlcNAc and yielded 72 mg of sialoside. For the biosynthesis of the non-deuterated sialosides, D_2_O was replaced with H_2_O, yielding 59 mg for X-Gal-α-2,3-Neu5Ac and 69 mg for X-Gal-α-2,3-Neu5Gc.

### 4.4. Analysis of the Sialosides by NMR Spectroscopy

Conventional ^1^H- and ^13^C-NMR spectra and 2-dimensional COSY and HSQC spectra (Figures S1–S16 in the Supporting Information) were acquired by using a 400 MHz NMR spectrometer (Avance AV400, Bruker, Karlsruhe, Germany) fitted with a 5 mm PABBO BB-1H/D Z-GRD probe. All experiments were carried out using 10 mg sialosides in 500 μL deuterated methanol. The residual solvent signal was used as the internal standard. Data were processed in MestReNova version 8.1 (Santiago de Compostela, Spain).

### 4.5. Analysis of the Sialosides by HPLC-ESI-MS

The analysis of generated sialosides was carried out using a Shimadzu LCMS-2020 system (Shimadzu Corporation, Kyoto, Japan), consisting of an LC-30AD pump, a SIL-30AC autosampler, and an SPD-20A UV detector unit (set at 300 nm), which was connected to an electrospray ionization (ESI) mass spectrometer. The analytes were separated using a reversed phase column (Hyperclone 5 µm ODS 120 Å, 250 × 4.60 mm, Phenomenex, Torrance, CA, USA). The mobile phases applied were 50 mM NH_4_COOH (pH 4.5) in water and acetonitrile for solvents A and B, respectively. A linear gradient of 10–60% B was applied from 0–5 min; then B was increased to 90% over 1 min and held at 90% for 2 min. B was then decreased to 10% in 1 min, and the column was equilibrated with 10% B for 6 min. The flow rate was 1 mL/min and the injection volume was 10 μL. The mass spectrometric analysis was performed in negative ion mode with a scan range from 400 to 1000 Da. Data were processed using the LabSolutions analysis software package (Shimadzu Corporation, Kyoto, Japan).

### 4.6. Acid Hydrolysis of d_3_-Sialosides and OPD Labeling

For quantitative sialic acid analysis, 100 mg aliquots of homogenized meat, boiled egg yolk or egg white samples were transferred into a 1.5 mL centrifugal tube, and aqueous acetic acid (1 mL, 2 M) was added. Then 50 μL of the component **9a** (for egg samples, concentration 100 μM) or **9b** (for meats, concentration 100 μM) was added. The mixture was incubated at 80 °C for 4 h before being centrifuged (18,000 g, 10 min, 4 °C). The supernatant was then dried by vacuum centrifugation, and the solid part then re-dissolved in H_2_O (20 µL). Then, 20 µL of OPD solution (10 mg/mL of OPD in a solution of 200 mM aqueous NaHSO_3_) was added and the derivatization mix incubated at 80 °C for 40 min in the dark. The samples were analyzed by reversed phase HPLC-ESI-MS as previously described by Cao et al. [33]. The MS instrument was operated in positive-ion mode, with selected *m*/*z* values of 404.0 Da (for the non-deuterated OPD-Neu5Ac), 407.0 Da (for deuterated OPD-Neu5Ac), 420.0 Da (for non-deuterated OPD-Neu5Gc) and 423.0 Da (for deuterated OPD-Neu5Gc).

## 5. Conclusions

In the presented study the enzymatic synthesis of two isotopically labeled sialic acid derivatives d_3_-X-Gal-α-2,3-Neu5Ac (**9a**) and d_3_-X-Gal-α-2,3-Neu5Gc (**9b**) was described and the compounds characterized using mass spectrometric and NMR-based methods. It was further demonstrated that these compounds can be used as internal standards for the absolute quantification sialic acids in meat and egg samples, and should be also applicable for the quantitative determination of sialic acids in other biological materials such as serum or tissue samples.

## Figures and Tables

**Figure 2:**
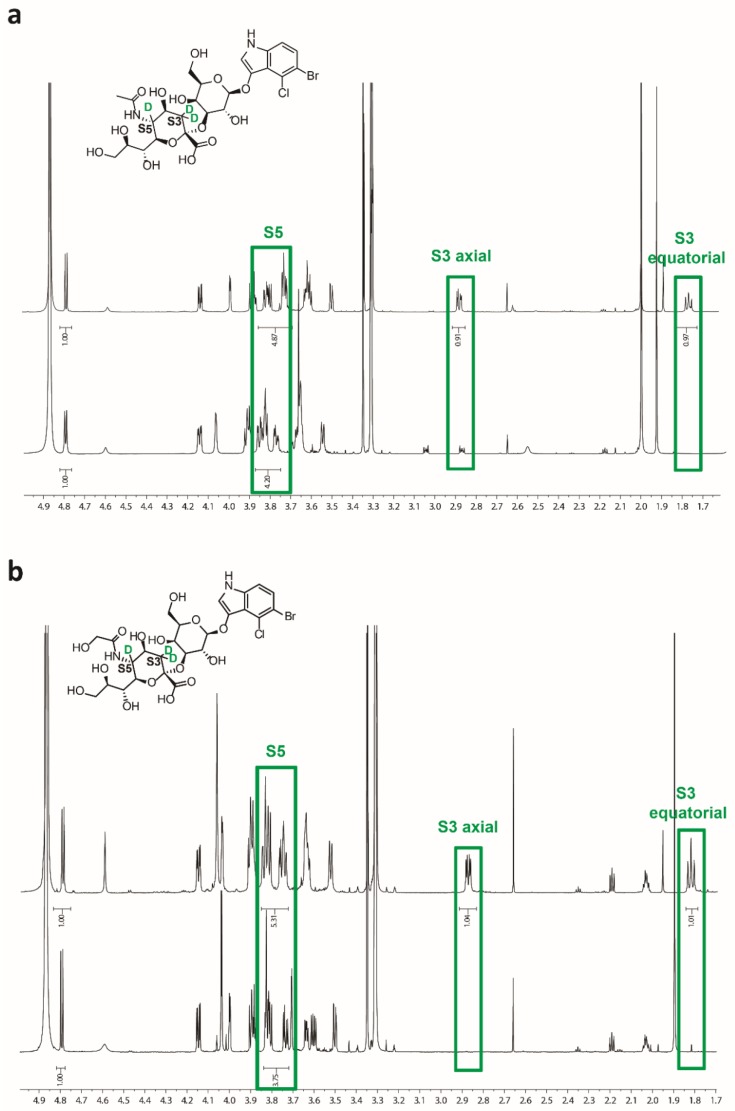
^1^H-NMR spectroscopic measurements of the *tri*-deuterated sialosides and nondeuterated sialosides: (**a**) X-Gal-α-2,3-Neu5Ac (top), compound **9a** (bottom), (**b**) X-Gal-α-2,3-Neu5Ac (top), compound **9b** (bottom).

**Table 1:** Neu5Gc concentrations in meat samples.

Species	Content (µg Neu5Gc/g Meat)
Beef	11.8 ± 3.8
Pork	5.7 ± 1.1
Wild boar	8.0 ± 0.8
Tibetan mutton	8.3 ± 1.0
Xu Zhou mutton	7.4 ± 2.3
Mongolian mutton	22.3 ± 4.0
Donkey	7.6 ± 6.0
Yak	8.2 ± 0.48

**Table 2:** Neu5Ac concentrations of egg white and egg yolk samples.

Species	Content (mg Neu5Ac/g Egg White and Egg Yolk)	
	Egg White	Egg Yolk
Chicken	0.40 ± 0.10	1.37 ± 0.15
Duck	0.18 ± 0.02	1.34 ± 0.13
Grey goose	0.05 ± 0.00	0.55 ± 0.06
Pigeon	1.50 ± 0.16	2.25 ± 0.13
Quail	0.23 ± 0.02	0.96 ± 0.11
Silkie chicken	0.26 ± 0.02	0.55 ± 0.05
Turkey	0.82 ± 0.11	1.15 ± 0.12
Guinea fowl	0.38 ± 0.06	0.98 ± 0.04

## Supplementary Materials

Supplementary materials are available online.
