# Supplementary material for: Enzymatic Synthesis of Trideuterated Sialosides
Source: Molecules. 2019 Apr 8;24(7):1368. doi: 10.3390/molecules24071368 (PMC6479850; doi:10.3390/molecules24071368)
Supplement: Supplementary file 1 [file molecules-24-01368-s001.pdf]

Supplementary Materials:

Enzymatic synthesis of tri-deuterated sialosides

Zhi P. Cai, Louis P. Conway, Ying Y. Huang, Wen J. Wang, Pedro Laborda, Ting Wang, Ai M. Lu, Hong L. Yao, Kun Huang, Sabine L. Flitsch, Li Liu and Josef Voglmeir

Table S1. NMR signals and correlations of X-Gal-Neu5Ac and tri-deuterated X-Gal-Neu5Ac (**9a**).

	**Non-deuterated X-Gal-Neu5Ac**				**Deuterated X-Gal-Neu5Ac**			
**Position**	**^{1}H**	**^{13}C**	**COSY**	**HMBC**	**^{1}H**	**^{13}C**	**COSY**	**HMBC**
**I2**	7.19 (s)	113.41	-	I7a,I3,I3a	7.19 (s)	113.47	-	I7a,I3,I3a
**I3**	-	138.51	-	-	-	138.43	-	-
**I3a**	-	119.56	-	-	-	119.57	-	-
**I4**	-	125.54	-	-	-	125.51	-	-
**I5**	-	113.95	-	-	-	114.09	-	-
**I6**	7.27 (d)	127.09	I7	I7a,I4	7.27 (d)	127.09	I7	I7a,I4
**I7**	7.12 (d)	112.61	I6	I5,I3a	7.12 (d)	112.61	I6	I5,I3a
**I7a**	-	135.06	-	-	-	135.08	-	-
**G1**	4.79 (d)	105.68	G2	I3,G5	4.79 (d)	105.64	G2	I3
**G2**	3.89 (m)	70.88	G1,G3	G1,G3	3.92 (m)	70.84	G1,G3	G1
**G3**	3.51 (dd)	69.60	G2	G4	3.54 (d)	62.26	G2	G4
**G4**	3.62 (m)	74.98	-	G3,G2	3.67 (m)	74.93	-	-
**G5**	3.62 (m)	77.07	G6	G6	3.67 (m)	76.63	G6	G6
**G6**	3.81 (m)	62.92	G5	G5	3.83 (m)	61.26	G5	G5
	3.74 (m)				3.77 (dd)			
**S1**	-	175.51	-	-	-	175.57	-	-
**S2**	-	101.26	-	-	-	101.57	-	-
**S3**	2.88 (dd)	42.23	S4	S5,S4,S2	-	28.20	-	-
	1.78 (t)							
**S4**	3.74 (m)	70.15	S3	-	3.83 (m)	69.81	-	-
**S5**	3.74 (m)	54.05	-	S4	-	54.61	-	-
**S′5**	-	175.18	-	-	-	175.57	-	-
**S″5**	2.01 (s)	22.70	-	S′5	2.02 (s)	22.78	-	S′5
**S6**	4.14 (dd)	77.87	S7	S7,S2	4.14 (dd)	77.81	S7	S7,S2
**S7**	3.89 (m)	73.04	S6	S8	3.92 (m)	73.08	S6	-
**S8**	4.00 (d)	69.14	-	S7	4.06 (d)	67.56	-	-
**S9**	3.81 (m)	64.54	-	-	3.83 (m)	62.91	-	-
	3.62 (m)				3.67 (m)			

Table S2. NMR signals and correlations of X-Gal-Neu5Gc and tri-deuterated X-Gal-Neu5Gc (**9b**).

	**Non-deuterated X-Gal-Neu5Gc**				**Deuterated X-Gal-Neu5Gc**			
**Position**	**^{1}H**	**^{13}C**	**COSY**	**HMBC**	**^{1}H**	**^{13}C**	**COSY**	**HMBC**
**I2**	7.19 (s)	113.45	-	I7a,I3,I3a	7.19 (s)	113.41	-	I7a,I3,I3a
**I3**	-	138.49	-	-	-	138.56	-	-
**I3a**	-	119.59	-	-	-	119.59	-	-
**I4**	-	125.55	-	-	-	125.59	-	-
**I5**	-	114.02	-	-	-	113.91	-	-
**I6**	7.27 (d)	127.09	I7	I7a,I4	7.27 (d)	127.09	I7	I7a,I4
**I7**	7.12 (d)	112.61	I6	I5,I3a	7.12 (d)	112.61	I6	I5,I3a
**I7a**	-	135.08	-	-	-	135.08	-	-
**G1**	4.79 (d)	105.69	G2	I3	4.79 (s)	105.71	G2	I3
**G2**	3.90 (m)	70.86	G1,G3	G1	3.89 (m)	70.87	G1,G3	G1
**G3**	3.52 (d)	69.34	G2	G4,G2	3.50 (dd)	70.17	G2	G4
**G4**	3.75 (m)	74.71	-	-	3.70 (s)	74.67	-	G5,G3
**G5**	3.63 (m)	76.86	G6	G6	3.64 (dd)	77.10	G6	G6
**G6**	3.83 (m)	64.42	G5	-	3.82 (m)	62.95	G5	G5
	3.75 (m)				3.73 (dd)			
**S1**	-	177.24	-	-	-	180.39	-	-
**S2**	-	101.49	-	-	-	101.19	-	-
**S3**	2.87 (dd)	41.96	S4	S5,S4,S2	-	24.23	-	-
	1.82 (t)							
**S4**	3.90 (m)	70.11	S3	-	3.82 (m)	69.24	S5	S5
**S5**	3.83 (m)	53.81	S6	-	-	49.95	-	S4
**S′5**	-	175.64	-	-	-	177.31	-	-
**S′′5**	4.06 (s)	62.70	-	S′5	4.06 (s)	62.69	-	
**S6**	4.15 (dd)	77.85	S5,S7	S2	4.15 (dd)	77.90	S7	S2
**S7**	3.90 (m)	73.15	S6	S6	3.89 (m)	73.11	S6	S6
**S8**	4.04 (d)	69.34	-	S7,S6	4.04 (d)	69.14	-	-
**S9**	3.83 (m)	62.93	-	S8	3.82 (m)	64.63	-	-
	3.63 (m)				3.61 (dd)			

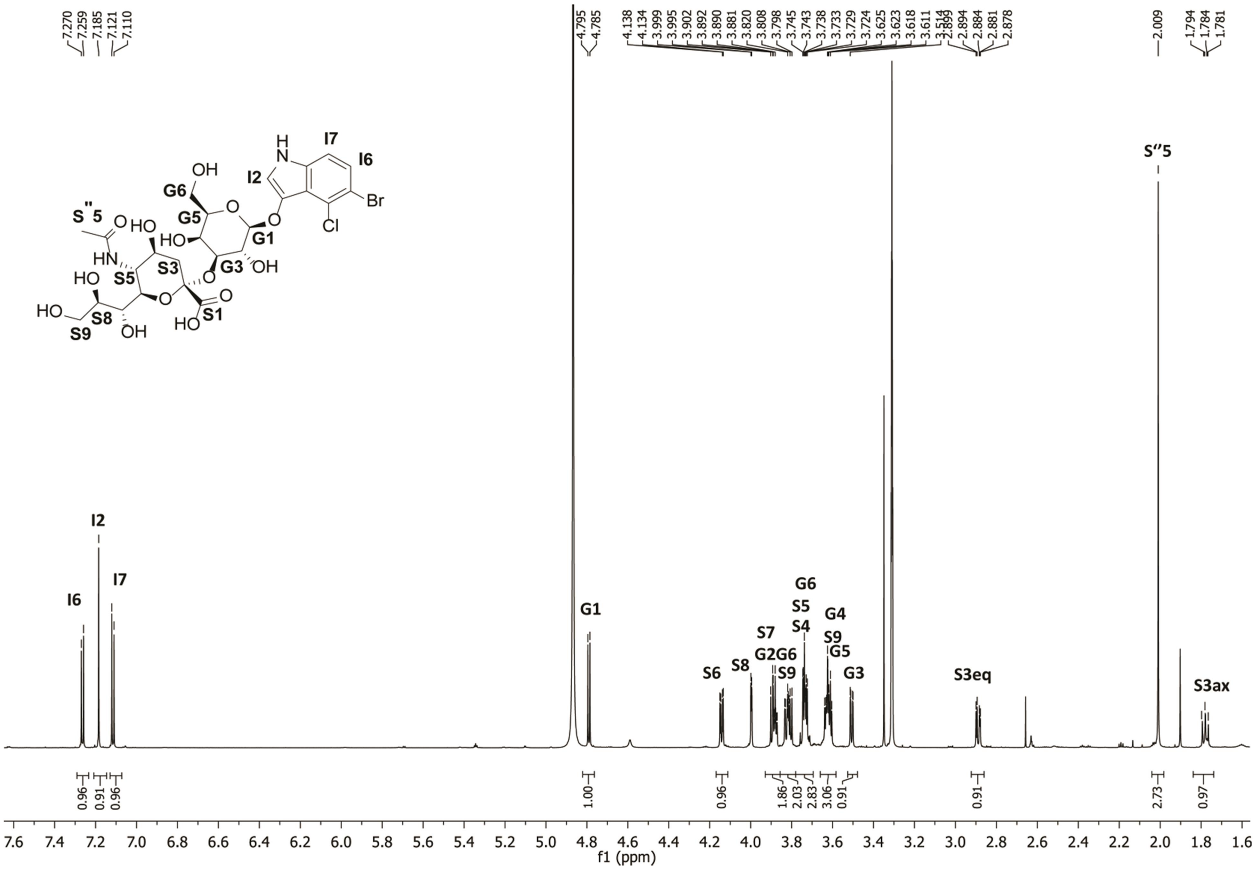

Figure S1. ^{1}H NMR spectrum of X-Gal-Neu5Ac. The spectrum was collected in a Bruker Avance AV400 using the deuterated methanol residual signal as internal standard.

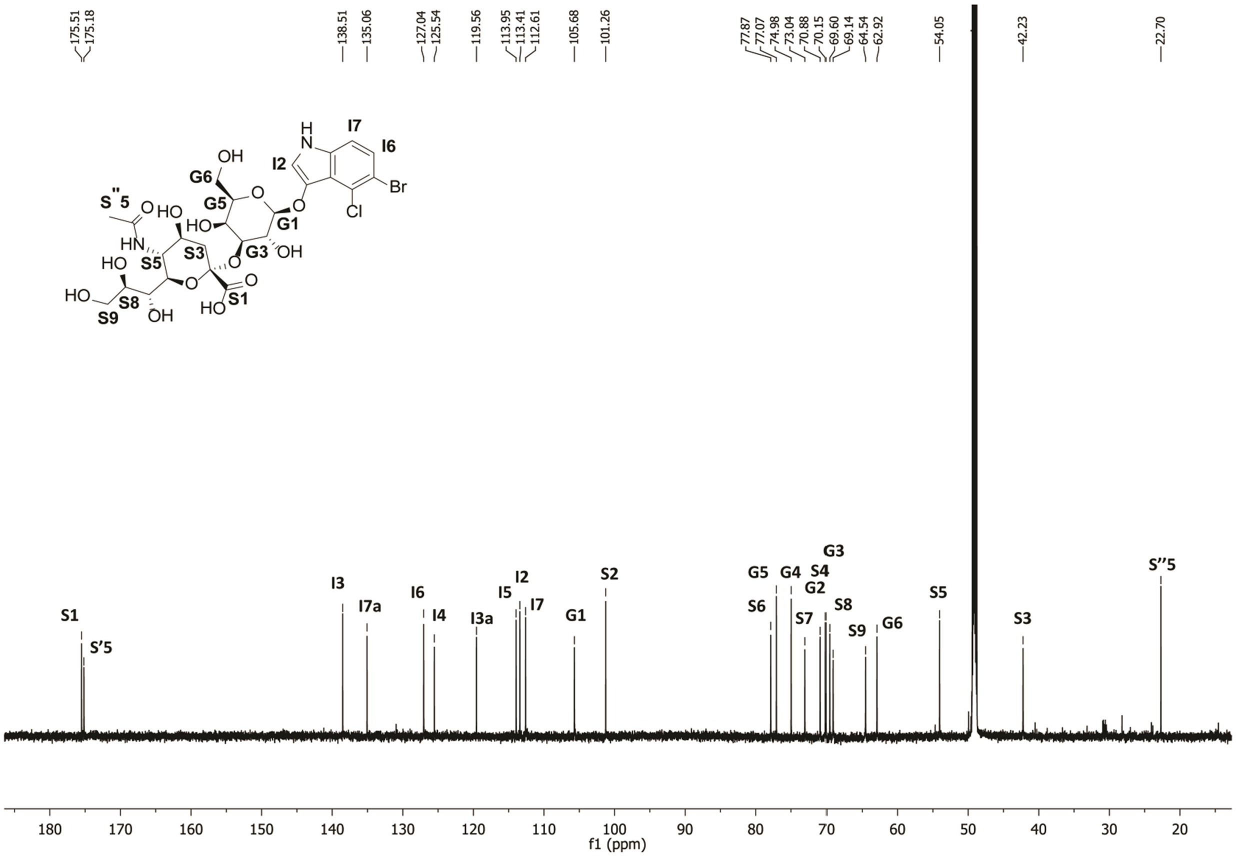

Figure S2. ^{13}C NMR spectrum of X-Gal-Neu5Ac. The spectrum was collected in a Bruker Avance AV400 using deuterated methanol residual signal as internal standard.

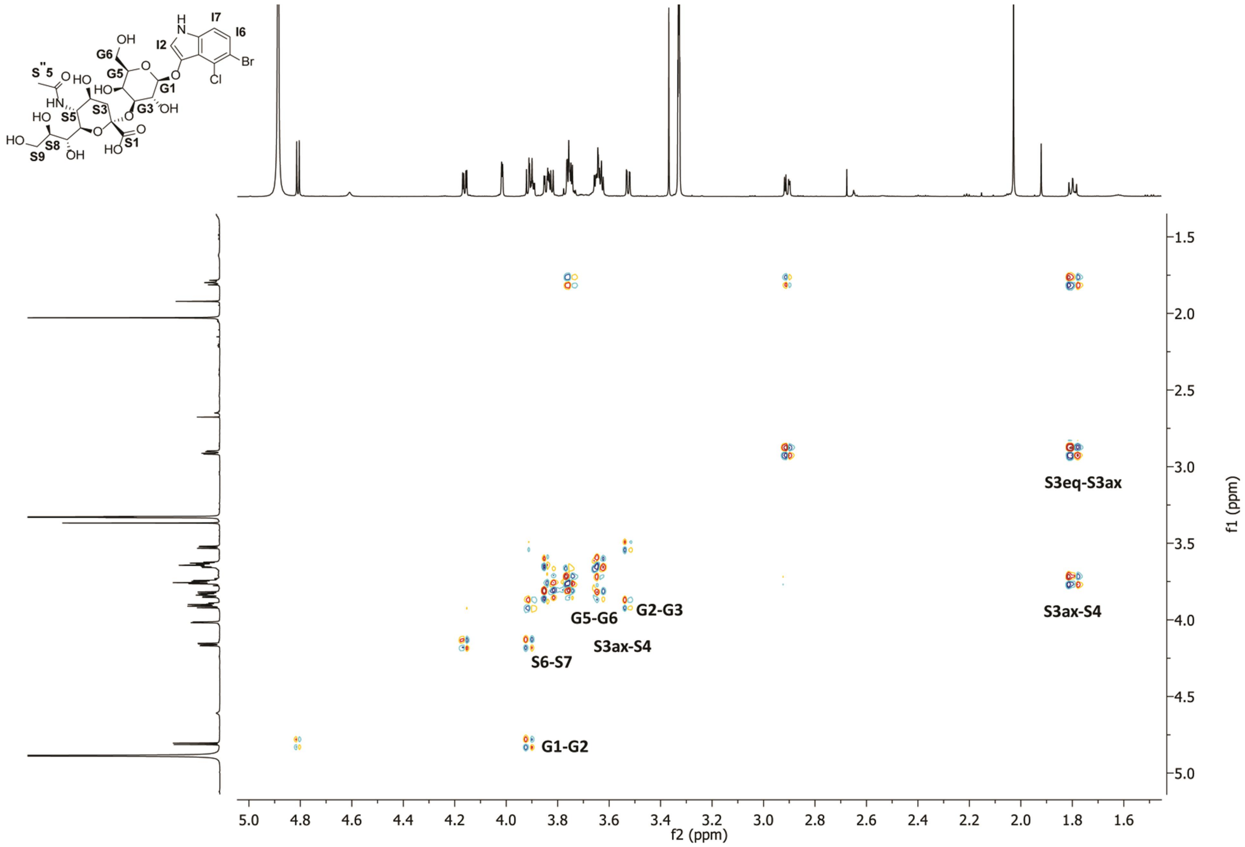

Figure S3. COSY NMR spectrum of X-Gal-Neu5Ac. The spectrum was collected in a Bruker Avance AV400 using deuterated methanol residual signal as internal standard.

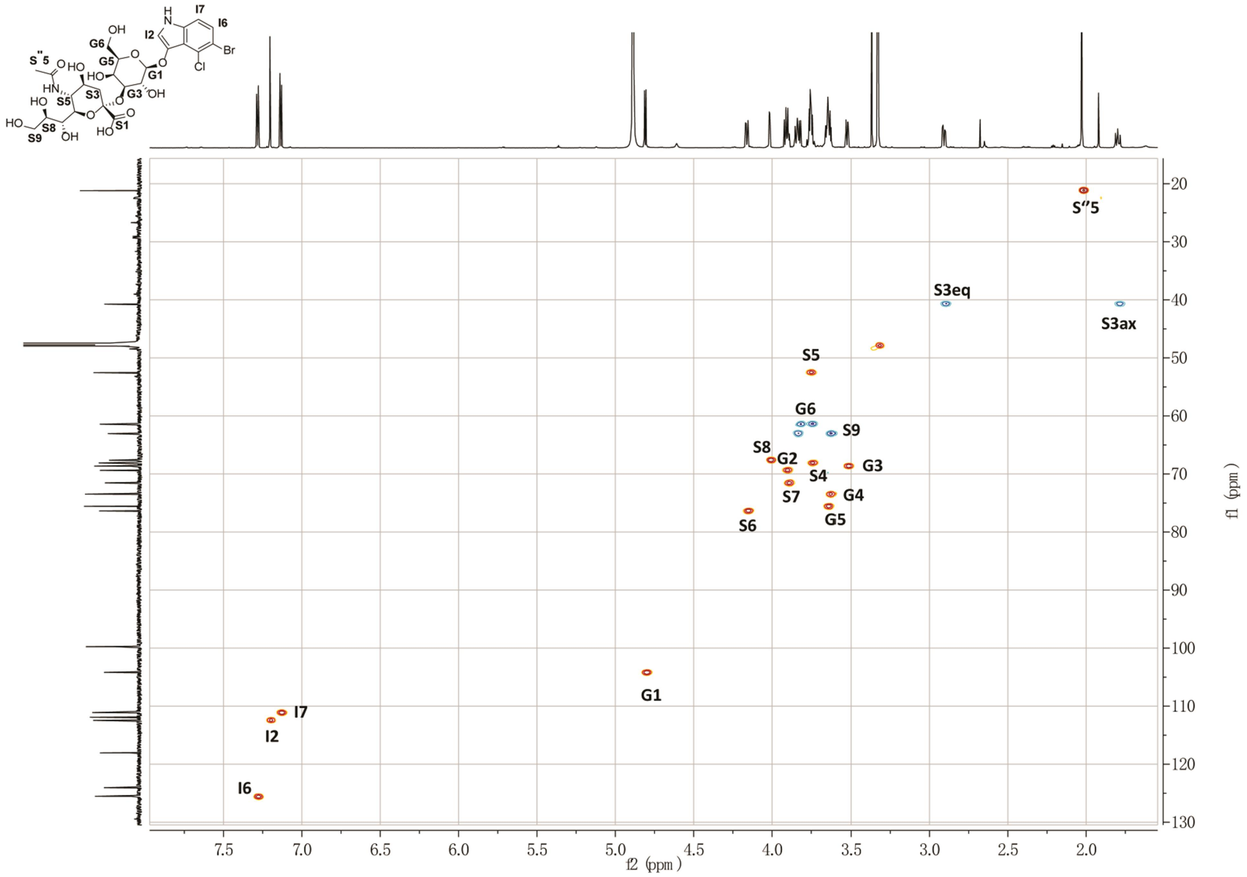

Figure S4. HSQC NMR spectrum of X-Gal-Neu5Ac. The spectrum was collected in a Bruker Avance AV400 using deuterated methanol residual signal as internal standard.

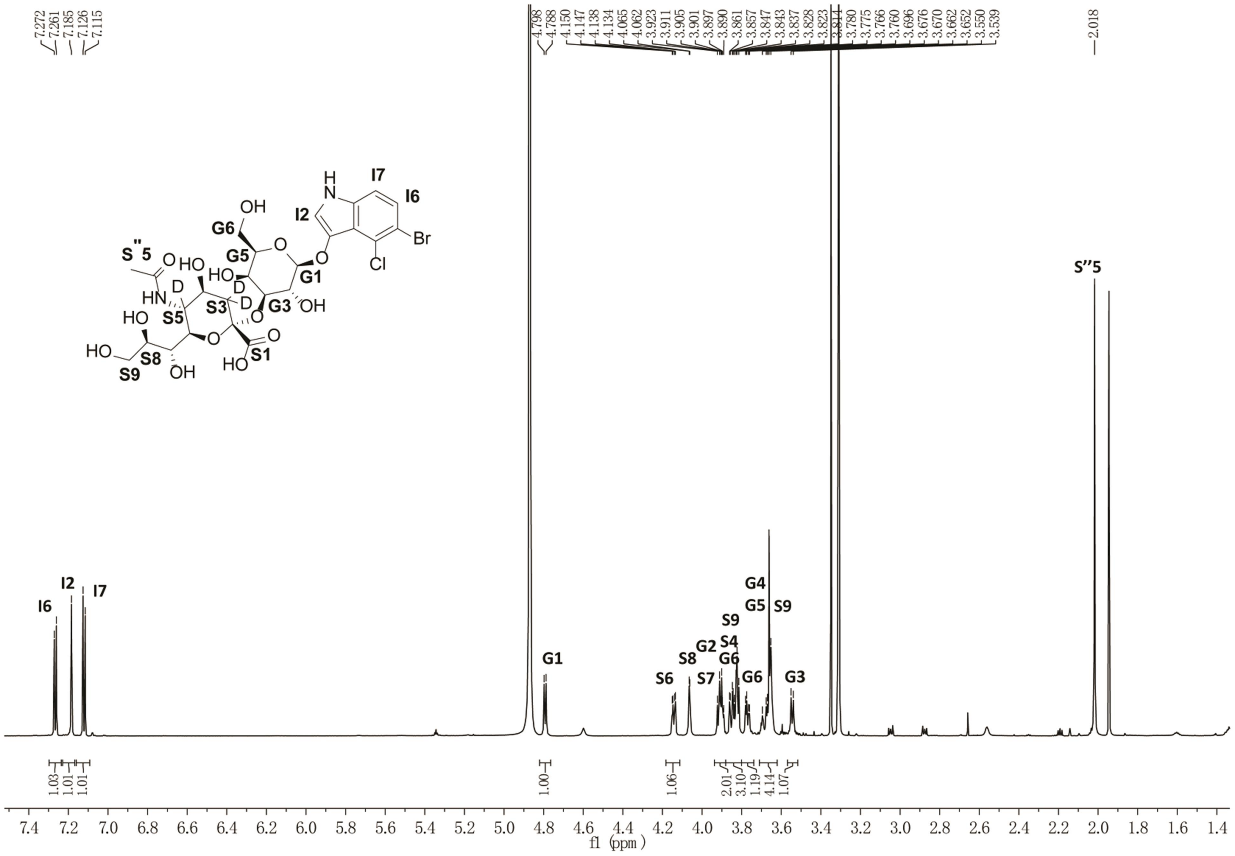

Figure S5. ^1H NMR spectrum of tri-deuterated X-Gal-Neu5Ac (**9a**). The spectrum was collected in a Bruker Avance AV400 using deuterated methanol residual signal as internal standard.

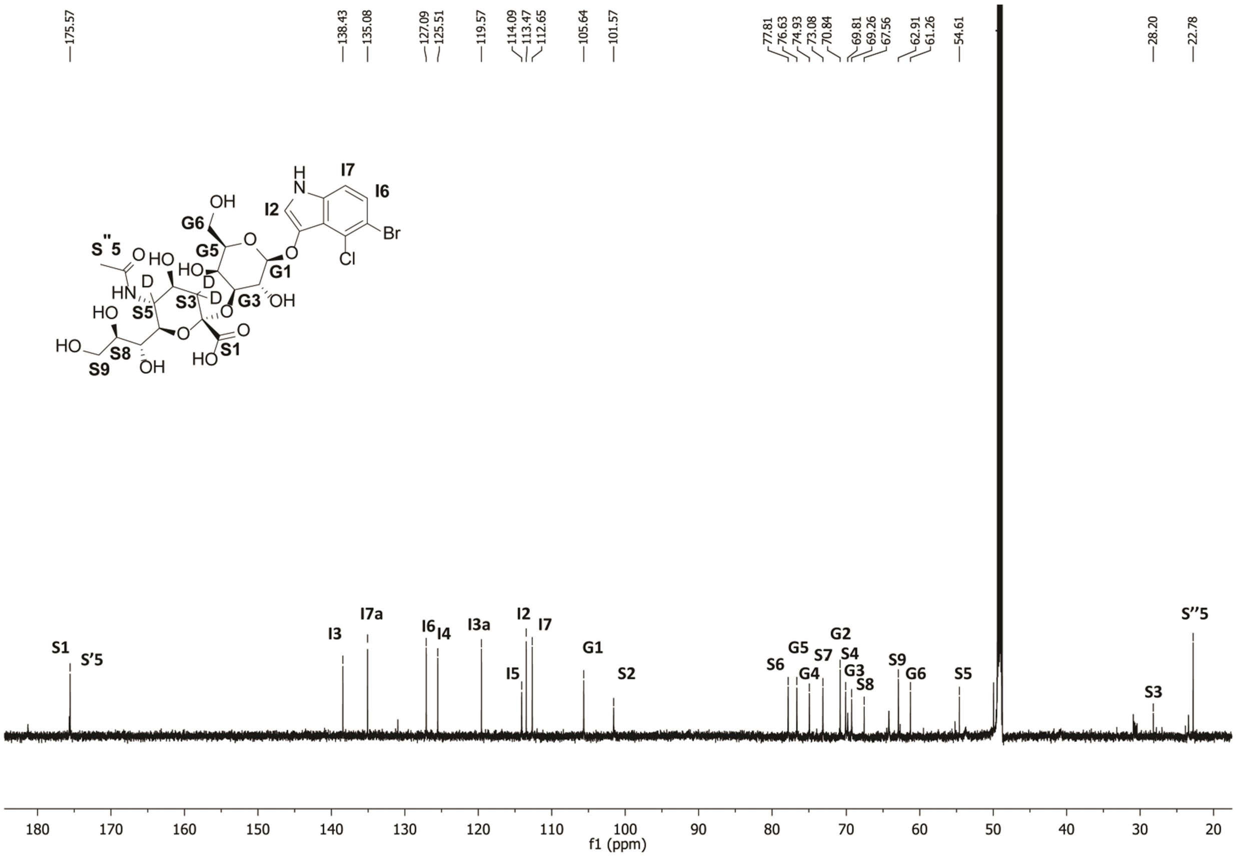

Figure S6. ^{13}C NMR spectrum of tri-deuterated X-Gal-Neu5Ac (**9a**). The spectrum was collected in a Bruker Avance AV400 using deuterated methanol residual signal as internal standard.

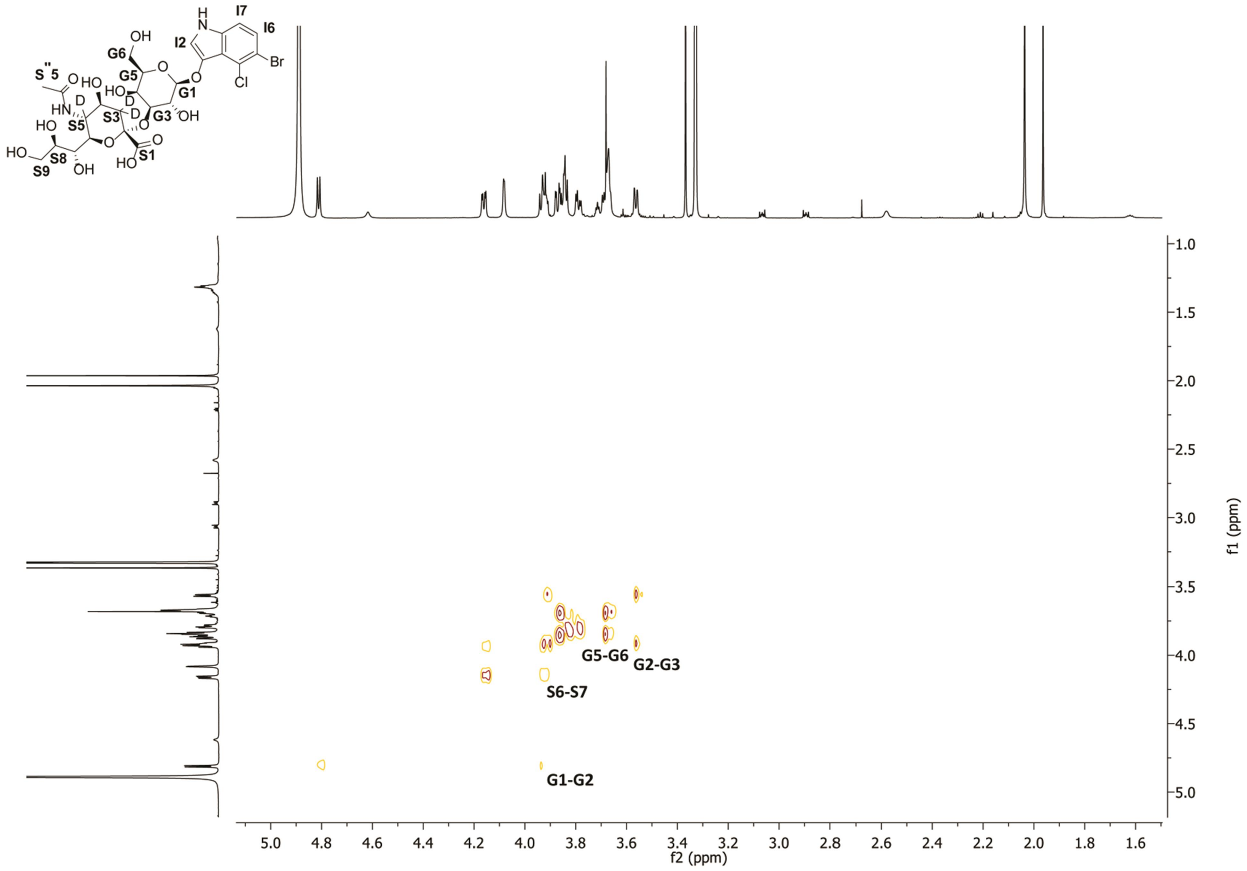

Figure S7. COSY NMR spectrum of tri-deuterated X-Gal-Neu5Ac (**9a**). The spectrum was collected in a Bruker Avance AV400 using deuterated methanol residual signal as internal standard.

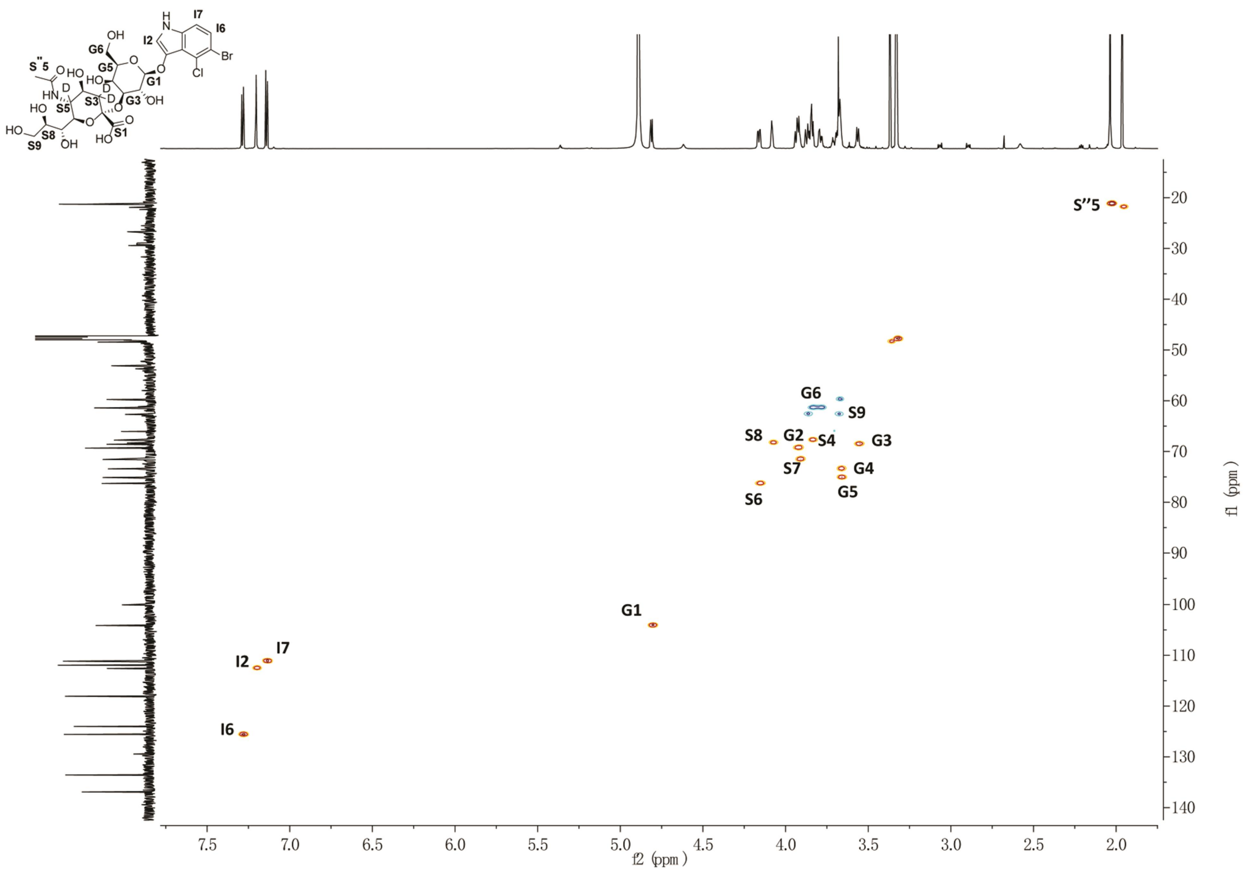

Figure S8. HSQC NMR spectrum of tri-deuterated X-Gal-Neu5Ac (**9a**). The spectrum was collected in a Bruker Avance AV400 using deuterated methanol residual signal as internal standard.

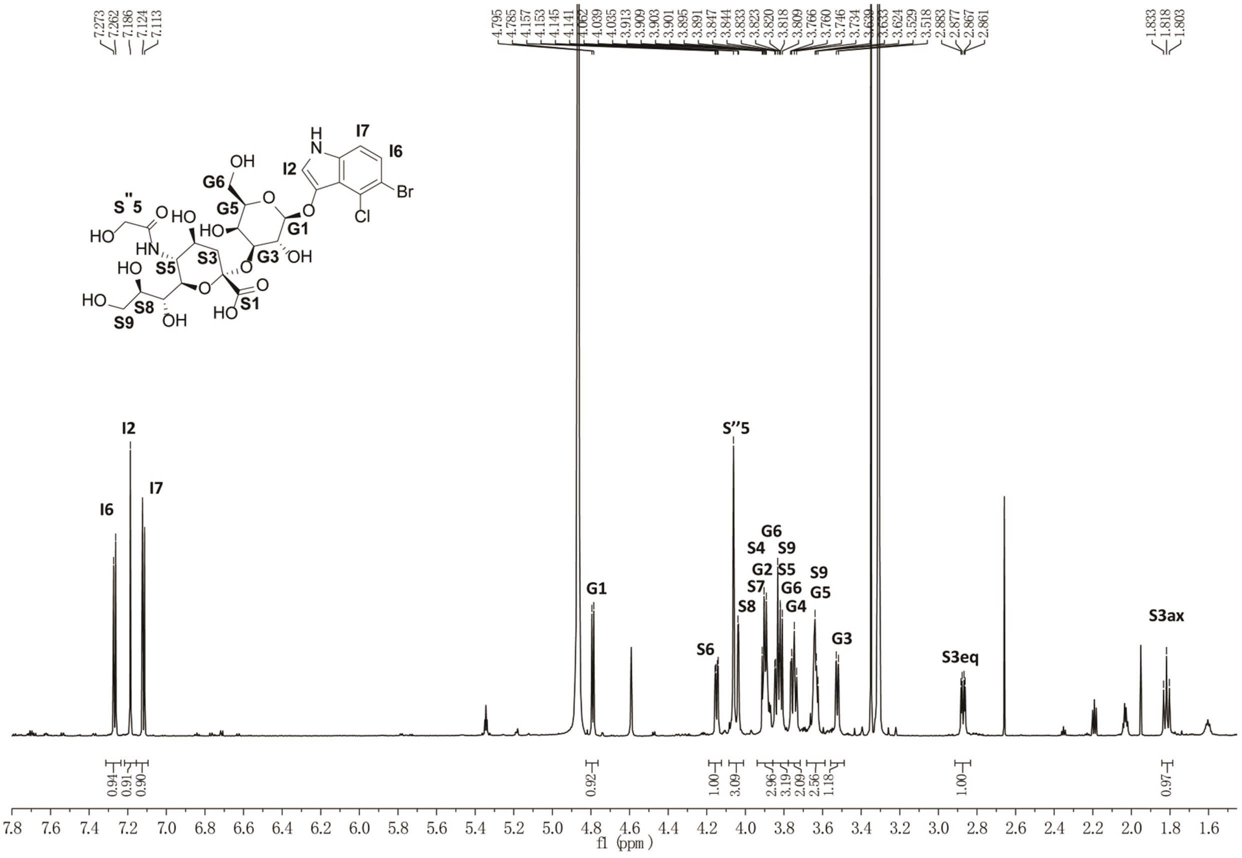

Figure S9. ^{1}H NMR spectrum of X-Gal-Neu5Gc. The spectrum was collected in a Bruker Avance AV400 using deuterated methanol residual signal as internal standard.

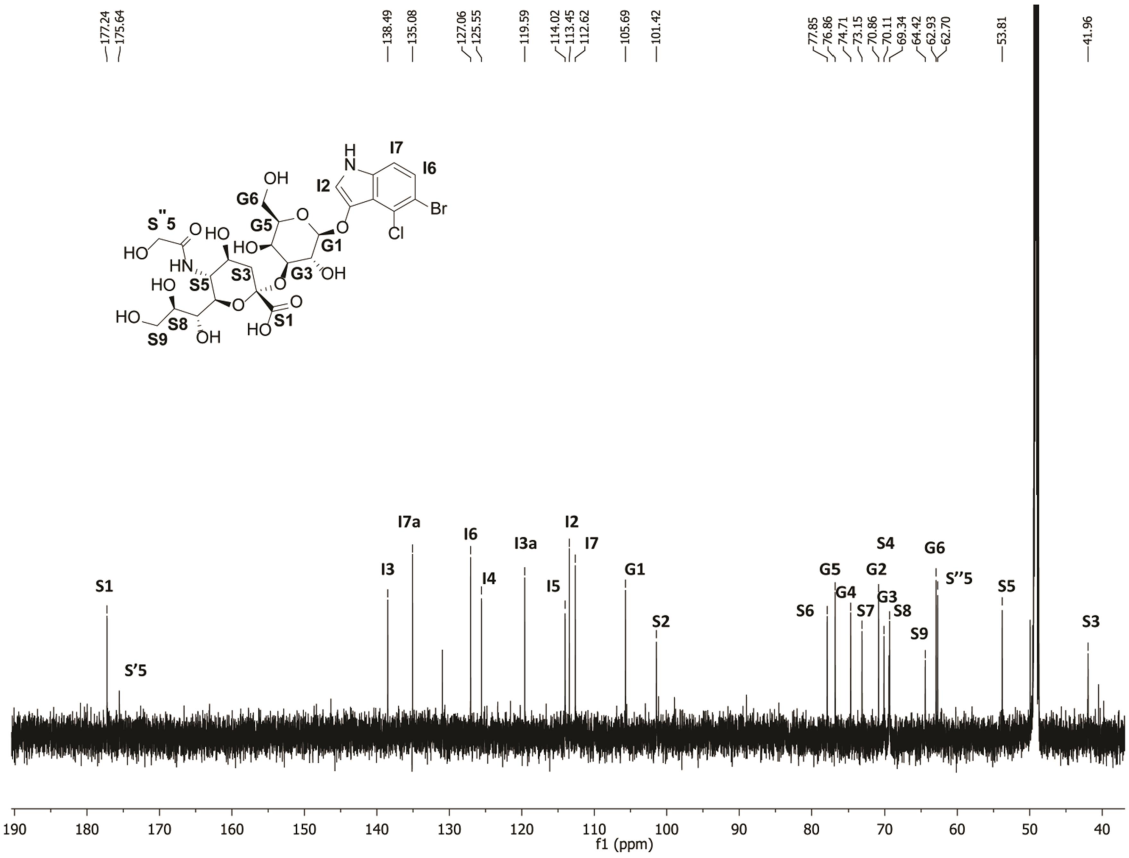

Figure S10. ^{13}C NMR spectrum of X-Gal-Neu5Gc. The spectrum was collected in a Bruker Avance AV400 using deuterated methanol residual signal as internal standard.

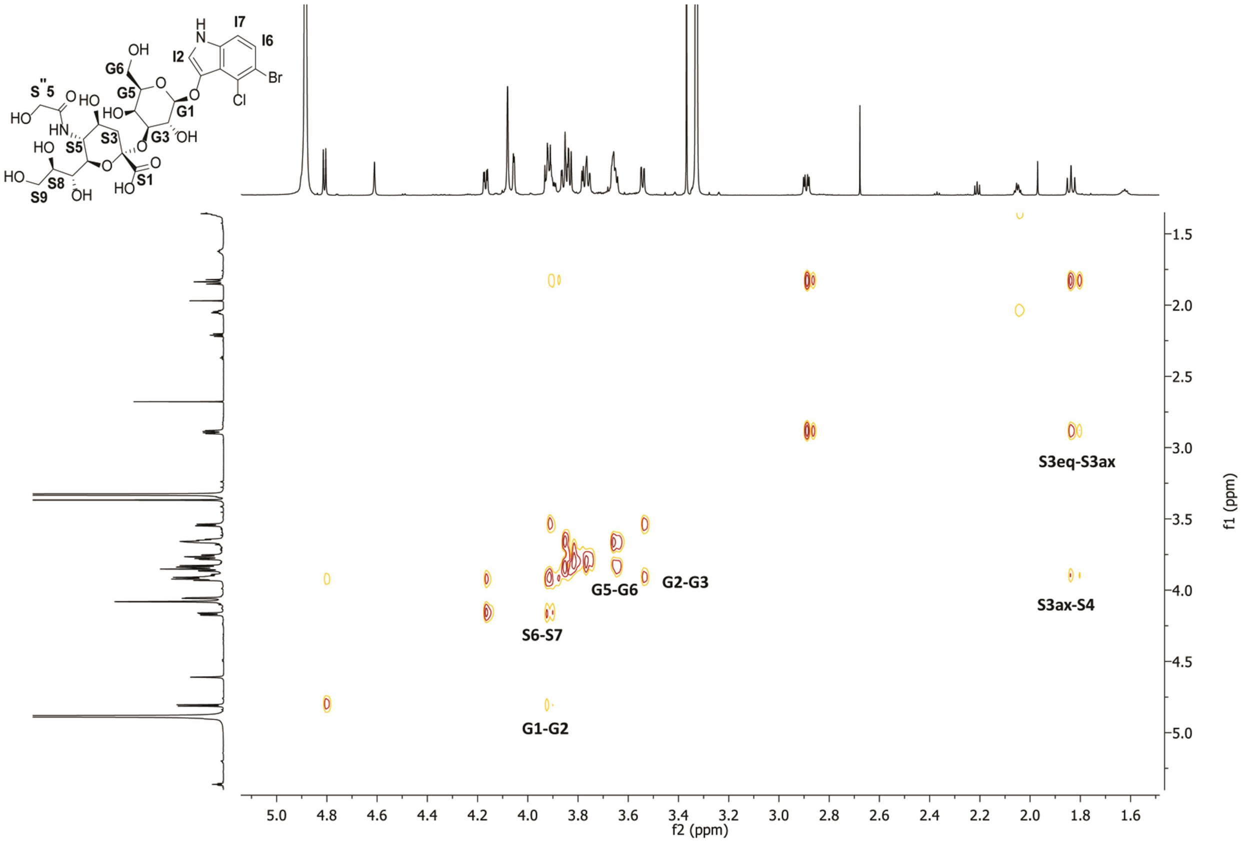

Figure S11. COSY NMR spectrum of X-Gal-Neu5Gc. The spectrum was collected in a Bruker Avance AV400 using deuterated methanol residual signal as internal standard.

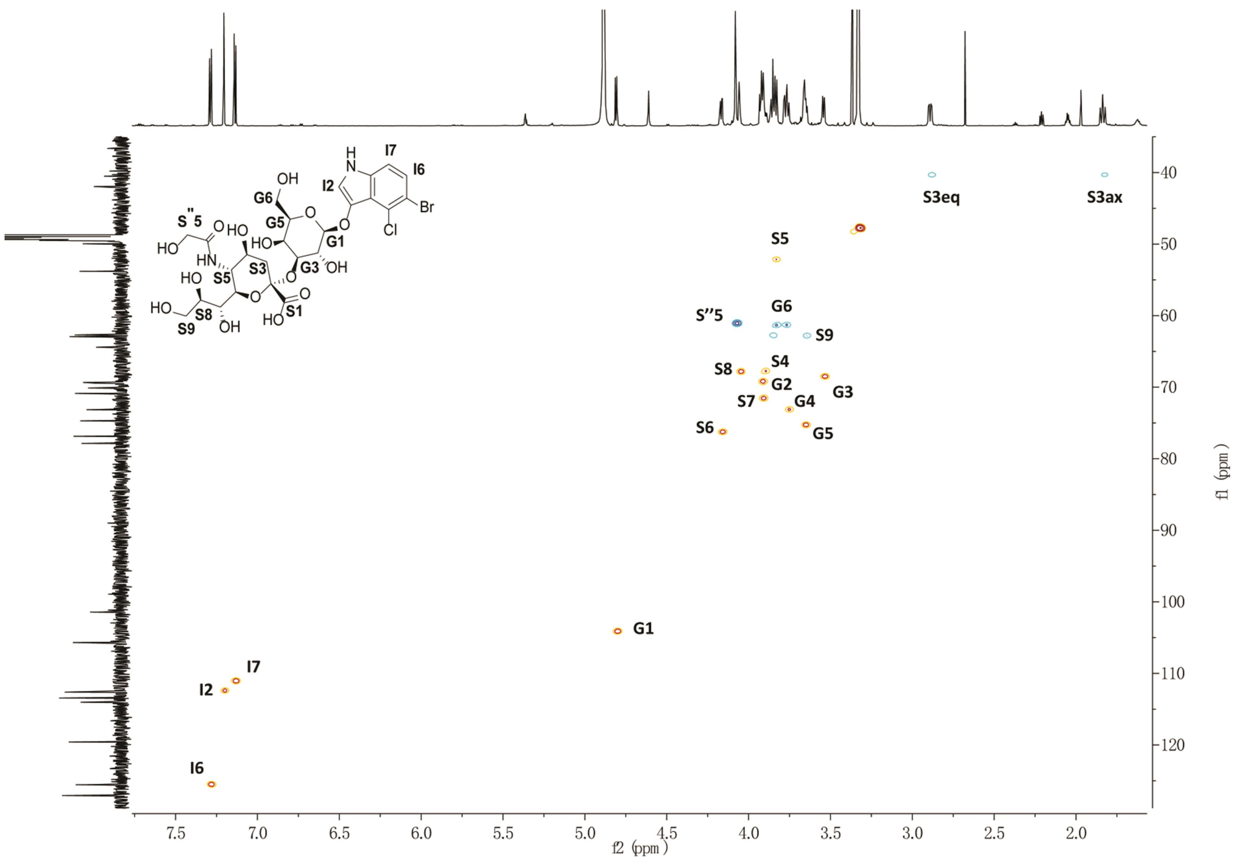

Figure S12. HSQC NMR spectrum of X-Gal-Neu5Gc. The spectrum was collected in a Bruker Avance AV400 using deuterated methanol residual signal as internal standard.

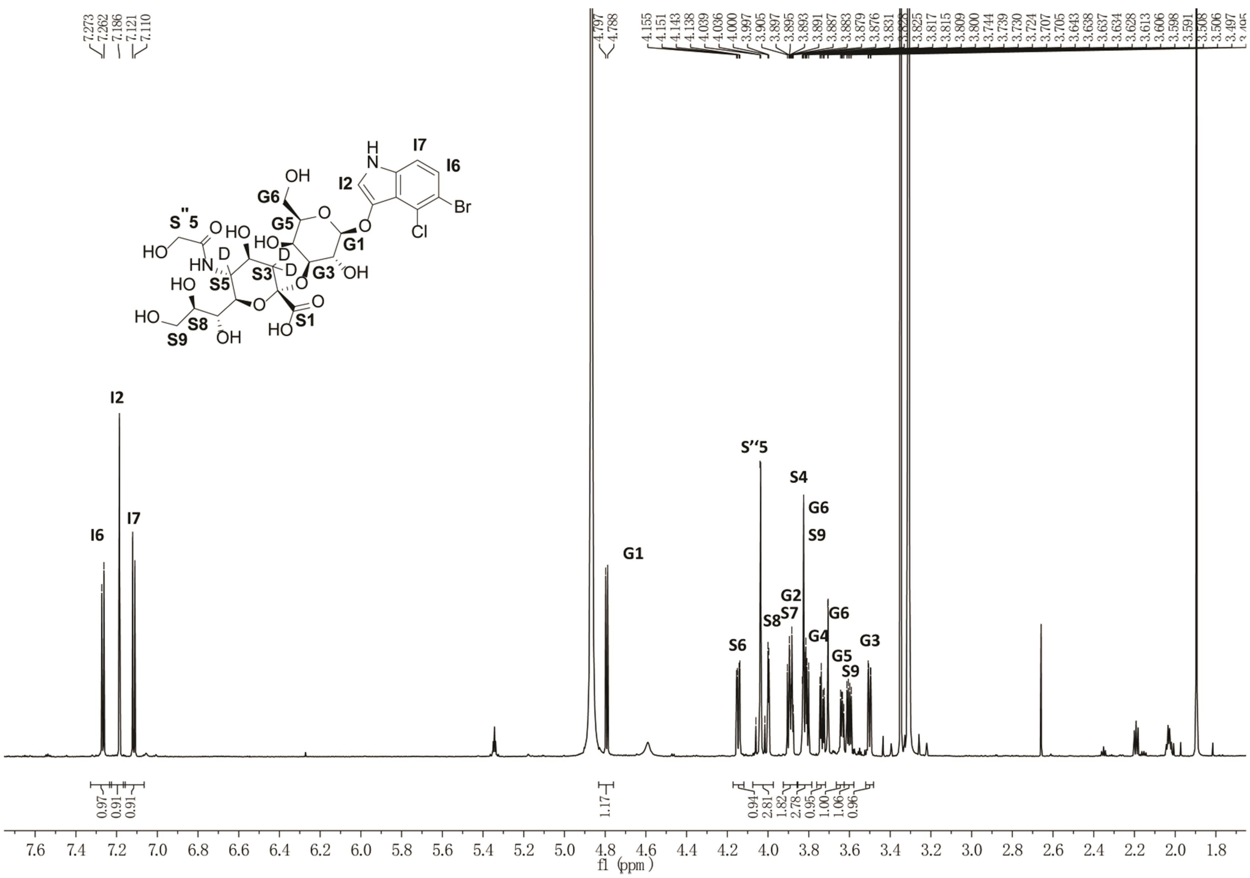

Figure S13. ¹H NMR spectrum of tri-deuterated X-Gal-Neu5Gc (**9b**). The spectrum was collected in a Bruker Avance AV400 using deuterated methanol residual signal as internal standard.

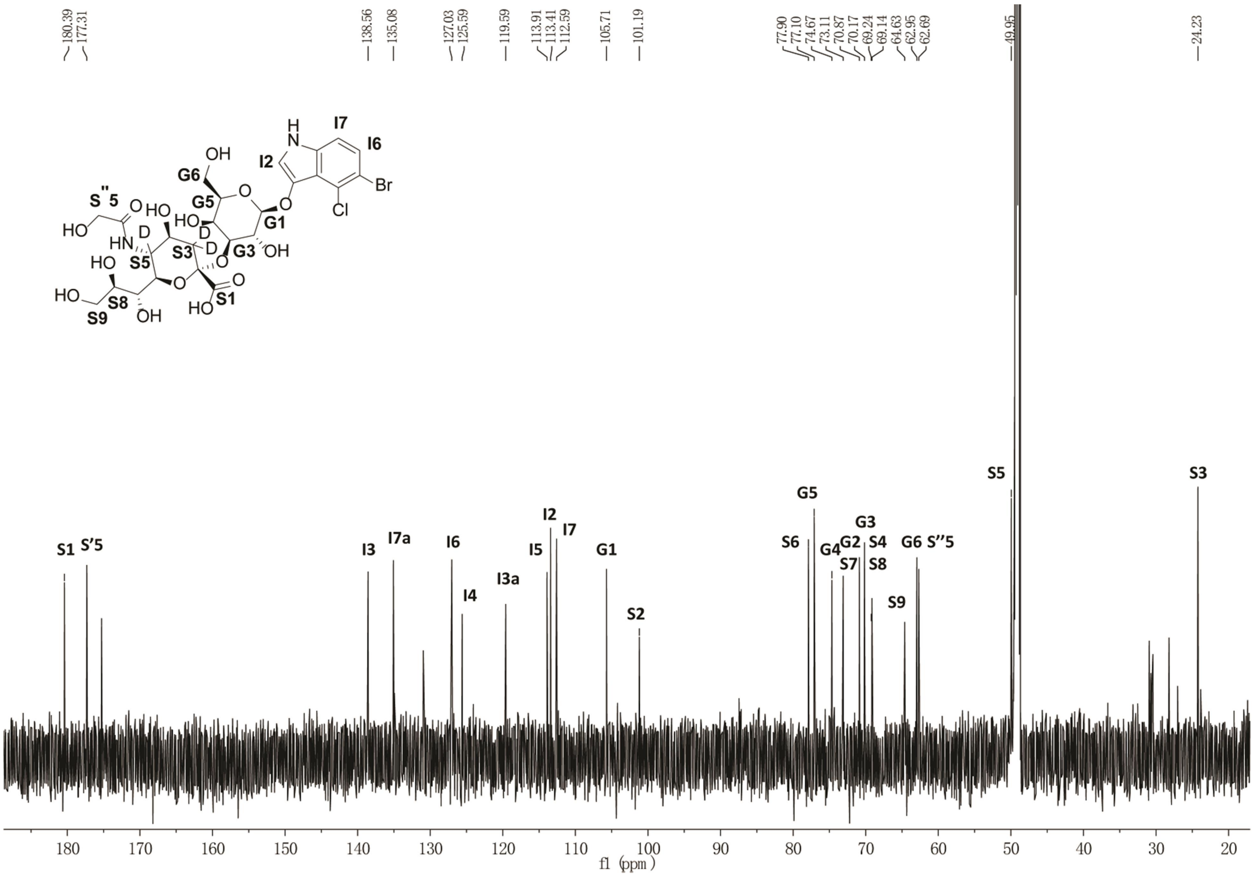

Figure S14. ^{13}C NMR spectrum of tri-deuterated X-Gal-Neu5Gc (**9b**). The spectrum was collected in a Bruker Avance AV400 using deuterated methanol residual signal as internal standard.

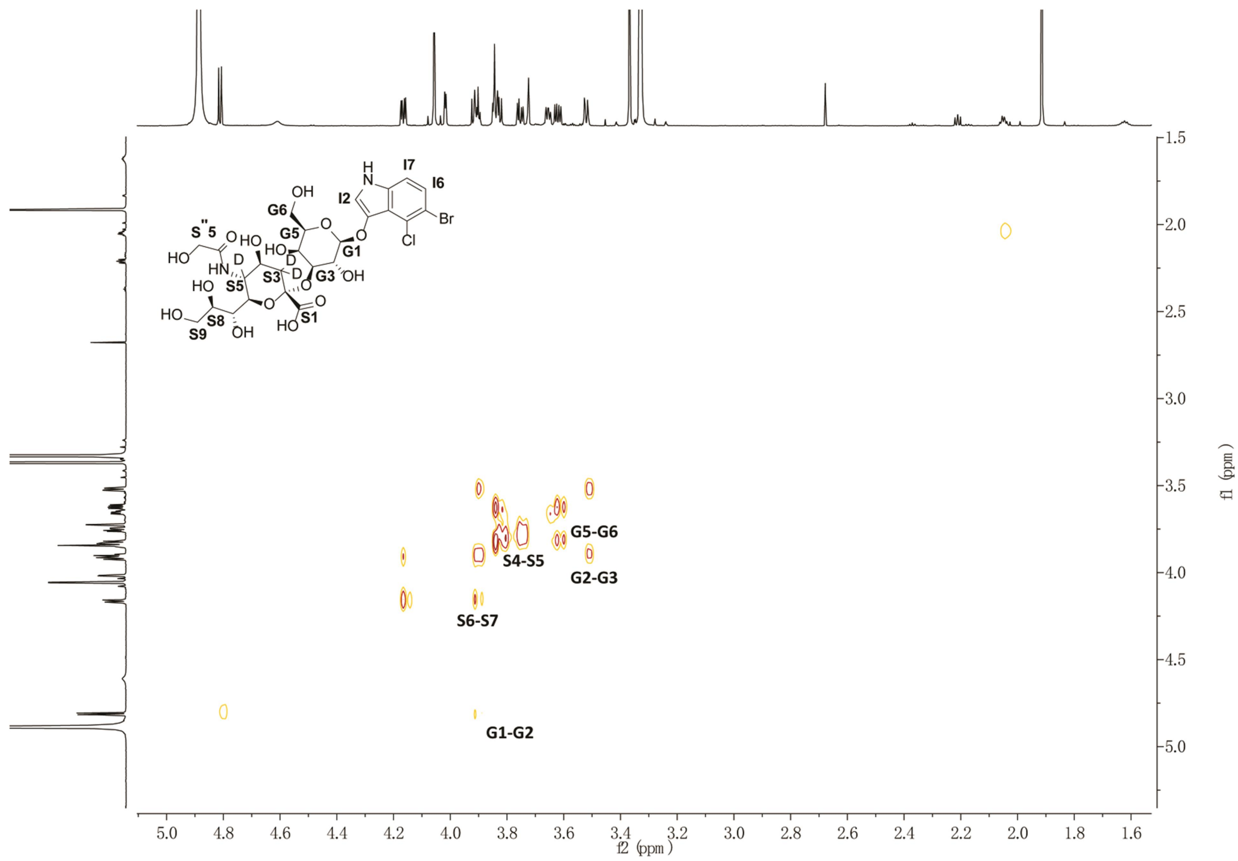

Figure S15. COSY NMR spectrum of tri-deuterated X-Gal-Neu5Gc (**9b**). The spectrum was collected in a Bruker Avance AV400 using deuterated methanol residual signal as internal standard.

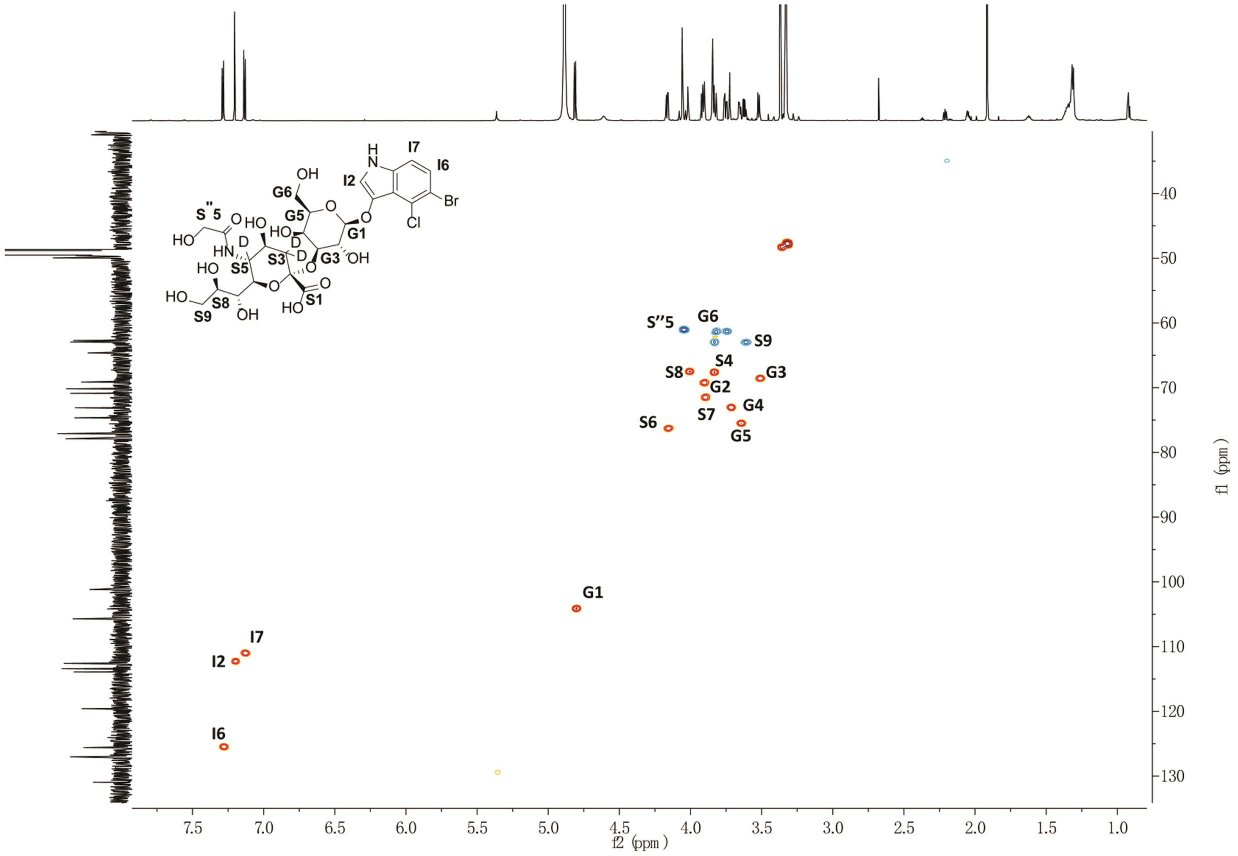

Figure S16. HSQC NMR spectrum of tri-deuterated X-Gal-Neu5Gc (**9b**). The spectrum was collected in a Bruker Avance AV400 using deuterated methanol residual signal as internal standard.
